# Supplementary material for: Codonoblepharonteae, a New Major Lineage among Orthotrichoideae (Orthotrichaceae, Bryophyta)
Source: Plants (Basel). 2022 Dec 16;11(24):3557. doi: 10.3390/plants11243557 (PMC9781468; doi:10.3390/plants11243557)
Supplement: Supplementary file 1 [file plants-11-03557-s001.zip › plants-2080396-supplementary.pdf]

Table S1. Samples information. Those in italics were newly sequenced for this study.

Taxon	ID	Voucher	Locality	Collector(s)	*ITS*2	*rps*4	*trn*G	*trnL*-F
Atlantichella calvescens (Carrington) F.Lara, Garilleti & Draper	ID199	MAUAM 4182	Spain, Canary Islands	F. Lara	MW264111	MW401337	MW401424	MW401501
Atlantichella calvescens (Carrington) F.Lara, Garilleti & Draper	ID400	MAUAM 4442	Spain, Asturias	B. Estébanez	MW264114	MW401340	MW401427	MW401504
Australoria chilensis (Calabrese & F. Lara) F.Lara, Garilleti & Draper	FL013	MAUAM 3767	Argentina, Neuquén	F. Lara & R. Garilleti	*OP508358*	*OP487613*	*OP487640*	*OP487660*
Codonoblepharon forsteri (Dicks.) Goffinet	ID384	MAUAM 1950	Spain, Álava	B. Albertos et al.	MW264075	MW401280	MW401362	MW401442
Codonoblepharon forsteri (Dicks.) Goffinet	ID397	MAUAM 4956	Spain, Málaga	F. Lara	MN529593	MN596378	MN542450	MN542426
Codonoblepharon forsteri (Dicks.) Goffinet	PAR011	MAUAM 3774	Spain, Málaga	G. Terroba & J. Terroba	-	*OP487614*	*OP487641*	*OP487661*
Codonoblepharon forsteri (Dicks.) Goffinet	PAR018	MAUAM 3778	Spain, Málaga	G. Terroba	*OP508359*	*OP487615*	*OP487642*	*OP487662*
Codonoblepharon forsteri (Dicks.) Goffinet	PAR020	MAUAM 3780	Spain, Málaga	G. Terroba	*OP508360*	*OP487616*	*OP487643*	*OP487663*
Codonoblepharon forsteri (Dicks.) Goffinet	PAR023	MAUAM 3782	France, Hérault	V. Hugonnot	*OP508361*	*OP487617*	*OP487644*	*OP487664*
Codonoblepharon forsteri (Dicks.) Goffinet	PAR024	MAUAM 3783	France, Cantal	V. Hugonnot	*OP508362*	*OP487618*	*OP487645*	*OP487665*
Codonoblepharon forsteri (Dicks.) Goffinet	PAR025	MAUAM 3784	France, Corse	V. Hugonnot	-	*OP487619*	*OP487646*	*OP487666*
Codonoblepharon menziesii Schwägr.	PAR026	CAS 34885	USA, California	J.R. Shevock	-	*OP487623*	-	*OP487670*
Codonoblepharon menziesii Schwägr.	PAR027	CAS 33741	USA, California	J.R. Shevock	-	*OP487624*	-	*OP487671*
Codonoblepharon menziesii Schwägr.	PAR028	CAS 33550	Australia, Southern Forest Region	J.R. Shevock & W.R. Buck	-	*OP487625*	-	*OP487672*
Codonoblepharon menziesii Schwägr.	PAR002b	MAUAM 3395	Australia, Macquarie Island	L. Pertierra	*OP508363*	*OP487620*	-	*OP487667*

Codonoblepharon menziesii Schwägr.	PAR003b	MAUAM 2975	New Zealand, North Island	F. Lara & E. San Miguel	-	*OP487621*	-	*OP487668*
Codonoblepharon menziesii Schwägr.	PAR005	MAUAM 3765	New Zealand, South Island	F. Lara & R. Garilleti	*OP508364*	*OP487622*	-	*OP487669*
Codonoblepharon menziesii Schwägr.	1603	BG 18773		Shevock	-	MW401281	MW401363	MW401443
Codonoblepharon menziesii var. *angustifolium* (Malta) Matcham & O'Shea	PAR037b	MAUAM 3828	New Zealand, North Island	F. Lara & R. Garilleti	*OP508365*	*OP487626*	*OP487647*	*OP487673*
Codonoblepharon minutum (Müll.Hal. & Hampe) Matcham & O'Shea	PAR009	MAUAM 3772	New Zealand, South Island	F. Lara & R. Garilleti	-	*OP487627*	-	*OP487674*
Codonoblepharon minutum (Müll.Hal. & Hampe) Matcham & O'Shea	PAR012	MAUAM 3775	New Zealand, South Island	F. Lara & R. Garilleti	*OP508366*	*OP487628*	*OP487648*	*OP487675*
Codonoblepharon pungens (Müll.Hal.) A.Jaeger	LaFarge-England 8097	Herb. Goffinet LaFarge-England 8097		LaFarge-England	-	AY618383	-	AY636005
Leratia obtusifolia (Hook.) Goffinet	798	Allen 12284		Allen	MW264076	MW401282	MW401364	MW401444
Lewinskya acuminata (H.Philib.) F.Lara, Garilleti & Goffinet	BV010	MAUAM 3272	Spain, Ávila	F. Lara	KT862263	KT862293	MN542451	KT862322
Lewinskya incana (Müll.Hal.) F.Lara, Garilleti & Goffinet	BV079	MAUAM 4576	Chile, Capitan Prat	J. Larraín	MN529598	MN177272	MN542455	MN542430
Lewinskya praemorsa (Venturi) F.Lara, Garilleti & Goffinet	BV060	MAUAM 4570	USA, Nevada	J.R.Shevock	MW264088	MN177265	MW401377	MW401456
Lewinskya shawii (Wilson) F.Lara, Garilleti & Goffinet	BV068	MAUAM 4587	Greece, Ipiros	F. Lara et al.	MW264093	MN177270	MW401379	MW401461
Lewinskya arborescens (Thér. & Naveau) F.Lara, Garilleti & Goffinet	BV070	MAUAM 4590	Tanzania, Kilimanjaro	F. Lara et al.	MN529595	MN177279	MN542452	MN542427
Macrocoma lycopodioides (Schwägr.) Vitt	BV024	MAUAM 2953	South Africa, Western Cape	F. Lara & E. San Miguel	KT804254	KT862288	KT804294	KT804333
Nyholmiella gymnostoma (Bruch ex Brid.) Holmen & E. Warncke	1219	NY 4809	Canada, Newfoundland and Labrador	Zander	-	MW401299	MW401387	MW401469
Nyholmiella obtusifolia (Brid.) Holmen & E.Warncke	O118	MAUAM 4343	Spain, Burgos	F. Lara	MN529599	JQ836797	JQ836901	JQ836986

Orthotrichum anomalum Hedw.	O120	MAUAM 4330	Spain, Asturias	B. Estébanez	MH275453	JQ836799	JQ836903	JQ836988
Orthotrichum callistomum Fisch.-Oost. ex Bruch & Schimp.	FL002	MAUAM 3403	Nepal, Sagarmatha National Park	F. Lara & I. Draper	MT902367	MW401300	MW401389	MW401470
Orthotrichum casasianum F.Lara, Garilleti & Mazimpaka	R398	MAUAM 1702	Spain, Álava	B. Albertos et al.	MN529600	JQ836811	JQ836915	JQ837000
Orthotrichum confusum R. Medina, F.Lara & Garilleti	R680	MAUAM 4323	USA, California	F. Lara et al.	MH275463	JQ836878	JQ836978	JQ837067
Orthotrichum persimile F.Lara, R.Medina & Garilleti	R580	UC-1650645	USA, California		MH275479	JQ836833	JQ836937	JQ837022
Pentastichella chilensis (Broth.) F.Lara, Garilleti & Draper	FL020	MAUAM 3787	Chile, Elqui	P. Drapela	*OP508367*	*OP487629*	*OP487649*	*OP487676*
Pentastichella chilensis (Broth.) F.Lara, Garilleti & Draper	FL021	JL 43034	Chile, Choapa	J. Larraín	*OP508368*	*OP487630*	*OP487650*	*OP487677*
Pentastichella pentasticha (Mont.) Müll.Hal. ex Thér.	FL022	MAUAM 3769	Chile, Aysén	F. Lara & R. Garilleti	*OP508369*	*OP487631*	*OP487651*	*OP487678*
Pentastichella pentasticha (Mont.) Müll.Hal. ex Thér.	ID207/BV038	MAUAM 2981	Argentina, Neuquén Villa La Angostura	F. Lara & E. San Miguel	KT862259	KT862289	KT804296	KT804335
Plenogemma phyllantha (Brid.) Sawicki, Plášek & Ochyra	ID331	MAUAM 5189	USA, Washington National Park	F. Lara, R. Garilleti & B. Albertos	KT804291	MW401330	KT804330	KT804370
Plenogemma phyllantha (Brid.) Sawicki, Plášek & Ochyra	ID387	MAUAM 2911	United Kingdom, England	F. Lara	MW264106	MW401332	MW401418	MW401497
Pulvigera lyellii (Hook. & Taylor) Plášek, Sawicki & Ochyra	BV018	MAUAM 4451	USA, California	F. Lara, V. Mazimpaka & B. Vigalondo	KT862282	KT862310	MN542458	KT862339
Pulvigera papillosa (Hampe) F.Lara, Draper & Garilleti	ID409	MAUAM 3477	Canada, British Columbia	F. Lara & R. Garilleti	MN529620	MN596395	MN542474	MN542446
Sehnemobryum paraguense (Besch.) Lewinsky & Hedenäs	1516	ALTA 21087	Brazil	Vitt	MW264109	AY618382	MW401421	AY636013
Stoneobryum bunyaense D.H. Norris & H.Rob.		US 00070599	Australia, Kiangaro Mt.		-	QBX99061	QBX99061	QBX99061
Stoneobryum mirum (Lewinsky) D.H.Norris & H. Rob.	1506	DUKE 156	South Africa, Eastern Cape	Vanderpoorten	MW264110	AY618381	MW401422	AY636012

Ulota coarctata (P.Beauv.) Hammar	ID360	MAUAM 4438	Spain, Cantabria	F. Lara et al.	KT804260	MN596397	KT804299	KT804338
Ulota crispula Bruch	ID355	MAUAM 4814	Ireland, Wicklow	B. Estébanez	KT804275	MN596398	KT804314	KT804353
Ulota drummondii (Hook. & Grev.) Brid.	ID356	MAUAM 5188	Japan, Hokkaido	R. Garilleti & F. Lara	KT804280	MW401344	KT804319	KT804359
Ulota macrodontia Dusén ex Malta	RG025	RG2016-236a	Chile, Aysén	R. Garilleti & F. Lara	MW264120	MW401348	MW401433	MW401510
Ulota streptodon Garilleti, Mazimpaka & F.Lara	RG022	RG2017-012e	Chile, Aysén	R. Garilleti & F. Lara	MW264124	MW401357	MW401437	MW401514
Zygodon campylophyllus Müll.Hal.	1128	DUKE 23100		Steere	MW264125	MW401358	-	MW401515
Zygodon catarinoi C.A.Garcia, F.Lara, Sérgio & Sim-Sim	PAR015	MAUAM 3385	Spain, Ciudad Real	F. Lara	*OP508370*	*OP487632*	*OP487652*	*OP487679*
Zygodon dentatus (Limpr.) Breidl. ex Gams	Zy1402	Kucera 17011	Czech Republic, Debrnik		MT921514	MT926369	-	MT926279
Zygodon fragilifolius Broth. ex Malta	FL018	MAUAM 3786	Tanzania, Mt. Kilimanjaro	F. Lara et al.	*OP508371*	*OP487633*	*OP487653*	*OP487680*
Zygodon hookeri var. *leptobolax* (Müll.Hal.) Calabrese	PAR007b	MAUAM 3771	New Zealand, South Island	F. Lara & R. Garilleti	*OP508372*	*OP487634*	*OP487654*	*OP487681*
Zygodon intermedius Bruch & Schimp.	PAR014	MAUAM 3777	New Zealand, South Island	F. Lara & R. Garilleti	-	*OP487635*	*OP487655*	*OP487682*
Zygodon peruvianus Sull.	1131	DUKE PV132		Griffin	MW264127	MW401360	MW401439	MW401517
Zygodon rupestris Schimp. ex Lorentz	PAR017	MAUAM 1893	Spain, Cádiz	F. Lara	*OP508373*	*OP487636*	*OP487656*	*OP487683*
Zygodon seriatus Thér. & Naveau	FL016	MAUAM 3785	Kenya, Mt. Kenya	J.A. Calleja et al.	*OP508374*	*OP487637*	*OP487657*	*OP487684*
Zygodon sibiricus Ignatov, Ignatova, Z.Iwats. & B.C.Tan	1183	MHA 1/82	Russia, Altai	Ignatov	-	MW401361	MW401440	MW401518
Zygodon stirtonii Schimp.	PAR022	MAUAM 4521	Spain, Cantabria	B. Estébanez	*OP508375*	*OP487638*	*OP487658*	*OP487685*
Zygodon trichomitrius Hook. & Wilson	PAR016	MAUAM 5000	Ethiopia, Kaffa	K. Hylander	*OP508376*	*OP487639*	*OP487659*	*OP487686*
Zygodon viridissimus (Dicks.) Brid.	ID208/BV0372	MAUAM 2910	United Kingdom, England	F. Lara	KT804258	KT862290	MW401441	KT862319
